# Establishment and application of a loop-mediated isothermal amplification method based on *MetAP2* gene for the detection of *Nosema bombycis* in silkworms (*Bombyx mori*)

**DOI:** 10.3389/fvets.2025.1549224

**Published:** 2025-03-10

**Authors:** Izhar Hyder Qazi, Ting Yuan, Sijia Yang, Christiana Angel, Jiping Liu

**Affiliations:** ^1^Guangdong Provincial Key Lab of Agro-Animal Genomics and Molecular Breeding, College of Animal Science, South China Agricultural University, Guangzhou, Guangdong, China; ^2^Key Laboratory for Agro-Ecological Processes in Subtropical Region, Institute of Subtropical Agriculture, The Chinese Academy of Sciences, Changsha, China; ^3^The University of Chinese Academy of Sciences, Beijing, China; ^4^Shaheed Benazir Bhutto University of Veterinary and Animal Sciences, Sakrand, Pakistan

**Keywords:** *Bombyx mori*, fumagillin, LAMP, molecular diagnosis, pebrine, sericulture

## Abstract

Pebrine, caused by *Nosema bombycis*, is a devastating disease of silkworms that causes huge economic losses to the sericulture farmers. Although pebrine is a quarantine disease, currently the development of effective molecular diagnostic or therapeutic tools for its management is still a lagging hotspot in sericulture research. In the present study, a highly specific, sensitive, and field-applicable LAMP assay targeting *MetAp2* gene was developed. LM1 primer set produced better results, with fluorescence (amplification) signals appearing in ~50 min. The reaction temperature of 60.9°C and outer primer to inner primer ratio of 1:8 were found to be optimal, with the shortest amplification time and strongest fluorescence intensity. The LAMP assay showed high specificity for the DNA of *Nosema bombycis* spores, as the templates of other common microorganisms of silkworms showed no amplification. The LAMP assay detected pMD-19T-met positive plasmid at the lowest concentration of 10^3^ copies, with a detection time of ~80 min. The practicality test showed that the LAMP assay can detect *Nosema bombycis* spore DNA at the lowest concentration of 10^−3^ ng/μL. At concentration of 1.2 ng/μL, the real-time fluorescence signals appeared in ~60 min. The LAMP assay detected *Nosema bombycis* at all life stages of untreated silkworms. In fumagillin treated silkworms, no real-time fluorescence amplification was observed at 90 h and later, indicating the reliability of LAMP in detecting *Nosema bombycis*, and effectiveness of fumagillin, to some degree, in treating pebrine infection. The developed LAMP assay holds good promise for its application as a specific and field-applicable tool for the detection/control of pebrine in the field settings.

## Introduction

Despite recent technological and industrial advancement, the global sericulture industry is still facing several economic challenges due to poor management and inadequate disease control strategies in production systems (1, 2). In particular, many bacterial, viral, fungal, and parasitic diseases directly affect the silkworm production in sericulture-intensive countries like India and China (3–5).

Microsporidia are a complex and hyper-diverse group of spore-forming intracellular pathogens that infect many vertebrate and invertebrate hosts, including agriculturally important insect species like honeybees, bumblebees, and wild and domestic silkworms (6–9). Pebrine is a very destructive microsporidian disease of silkworms that causes tremendous economic losses to the sericulture industry (4). *Nosema bombycis*, the causal pathogen of pebrine disease in the silkworm (*Bombyx mori* L.), was the first microsporidian species to be identified in 1857 (10, 11). However, despite its widespread occurrence and distribution, the diagnosis, prevention and treatment of pebrine are still a lagging hotspot in sericulture research (10).

*Nosema bombycis* has the ability to survive in harsh environment by forming mature dormant spores. It can infect silkworms though horizontal (fecal-oral) and vertical (transovarial) routes. Pebrine-infected silkworms show signs of delayed growth, impaired molting, lesions in the silk glands, peculiar black spots/patches on whole body, and eventual death (12–15). At the field level, the diagnosis and treatment of pebrine are very challenging due to the late appearance of obvious symptoms. It becomes more challenging when infection is asymptomatic and mild, making it deceptive and difficult to diagnose during routine inspection. Due to these reasons, pebrine remains the only disease with a mandatory quarantine (4) in the sericulture industry.

Due to transovarial transmission of *Nosema bombycis* from mother to progeny, the screening of the mother moths after egg laying is practiced in the silkworm rearing systems (16). Unfortunately, even today, the 150-years-old mother moth microscopic examination is still the preferred method to detect pebrine in silkworms. However, this method has inherent limitations like low specificity and sensitivity. In addition, skilled technicians with a high level of expertise (4) are required to differentiate between *Nosema bombycis* spores and that of other co-circulating non-pathogenic *Nosema* spp. and contaminating molds (16–18). Therefore, any human error during microscopic observation can produce false positive returns, leading to huge economic losses to farmers.

During the last two decades, a number of molecular techniques have been developed to identify and diagnose several silkworm pathogens, including *Nosema bombycis* [reviewed in (3)]. For instance, lately efforts have been made to develop field-friendly diagnostic methods for pebrine detection that are sensitive, labor-saving, and have the potential to practically replace the tedious microscopic examination method. Methods like immunodiagnostics (19, 20), nucleic acid lateral flow strip (21), mass spectrometry (18), conventional PCR (22–24), multiplex PCR (25), Taqman (reverse transcription PCR; 16), quantitative PCR (4, 26–29), and multiplex-crRNA CRISPR/Cas12a (30) assays have been developed to detect pebrine infection in wild and domestic silkworms. However, the practical use of these methods is still very limited due to their inherent limitations of low specificity, sensitivity, non-essential target genes, long procedural time, high cost, and need of sophisticated equipment and a high technical skill set.

The loop-mediated isothermal amplification (LAMP), a relatively new molecular technique, is used to amplify a limited amount of DNA into a million copies within an hour (31, 32). Although developed by Notomi et al. (33) in 2000, commercialization of the LAMP assay has been reported lately. The LAMP stands out an effective diagnostic test among PCR-based molecular techniques, primarily due to its sensitivity, specificity, low-cost, simplicity, rapidity, and practical application in various fields including medicine and agriculture (31, 32). Due to these significant characteristics, the LAMP fulfills all criteria proposed by the WHO on ideal diagnostics (32, 34).

In recent past, our group (35) and other researchers have made efforts in developing LAMP assays targeting different genes of *Nosema bombycis* (35–39). In our previous study (35), we developed a LAMP assay targeting *End binding 1* (*EB1*) gene of *Nosema bombycis* that was specifically focused on the detection of pebrine infection in the silkworm eggs. Meanwhile, the other three (37–39) of the previous reports were based on primer sets targeting the *small subunit ribosomal RNA* (*16S rRNA*) gene of *Nosema bombycis*. More importantly, there have been enough concerns over selection of *rRNA* gene sequences for the development of molecular diagnostic assays for the detection of microsporidian spp. (40, 41). These issues and other limitations are sufficiently elaborated in the discussion chapter of this article.

It has been argued that the essential genes of *Nosema bombycis* should be exploited to develop therapeutic and diagnostic options for managing pebrine disease of silkworms (26, 42). *Methionine aminopeptidase type 2* (*MetAP2*) is a protein coding gene of microsporidia that is essential for their survival. *MetAP2* is a highly validated target for treatment of microsporidiosis.^1^ Drugs such as fumagillin and its derivatives can irreversibly inhibit MetAP2 of microsporidia, leading to impaired protein homeostasis that is necessary for their survival (43–45). Importantly, although fumagillin was not found to be effective against all microsporidia spp. (14, 44), it is the only approved veterinary drug that has been used as an effective therapeutic agent to treat nosemosis in honeybees (14, 46, 47). Recently, we have reported that MetAP2 gene and amino acids sequences of *Nosema bombycis* (Guangdong isolate) shared a close evolutionary relationship with *Nosema* spp. of wild silkworms, but it was divergent from microsporidian spp. of other insects, *Aspergillus* spp., *Saccharomyces cerevisiae*, and higher animals including humans (48). In the present study, we used *MetAP2* gene as a target for the development of LAMP assay, because it is an essential protein coding gene and is present in a single copy in microsporidian genomes, including *Nosema bombycis* (42, 48). In addition, we believe that the LAMP assay based on *MetAp2* gene has a broader potential: it can be used for the detection of pebrine in silkworms, and can also be used to evaluate the effectiveness of fumagillin in treating *Nosema bombycis* infection in silkworms in the field.

To sum-up, herein we report the development and application of a specific, sensitive, and field-applicable LAMP assay targeted on *MetAp2* gene for the detection of pebrine in silkworms. In addition, a pilot experiment was conducted to test the practicality of the LAMP assay before and after fumagillin treatment in artificially infected silkworms. In doing so, we also evaluated the potential effectiveness of fumagillin in treating/limiting *Nosema bombycis* infection in domestic silkworms.

## Materials and methods

### Collection and purification of *Nosema bombycis*

*Nosema bombycis* spores were propagated and purified from silkworms (*Bombyx mori*) maintained in our laboratory as described in our previous report (48).

### Extraction of DNA

The DNA of *Nosema bombycis, Bacillus bombysepticus, Bacillus thuringiensis, Beauveria bassiana, Bombyx mori cytoplasmic polyhedrosis virus* (*BmCPV*), *Bombyx mori nuclear polyhedrosis virus* (*BmNPV*), *Bombyx mori densonucleosis virus* (*BmDNV*), and silkworms was extracted using the DNeasy Mini Kit (Qiagen, Germany). Note: the DNA of common pathogens of silkworms including *Bacillus bombysepticus, Bacillus thuringiensis, Beauveria bassiana, BmCPV, BmNPV, BmDNV* was used as control to evaluate the specificity of the LAMP primers.

### LAMP primer design based on target gene

The full-length sequence (1,278 bp) of *MetAP2* gene [(48); see Additional File 1 for full length sequence and GenBank accession ID: KX185053.1 for cds] of *Nosema bombycis* was used as a target for designing the LAMP primers. The specific LAMP primers were designed using the default feature of the Primer Explorer software (https://primerexplorer.jp/e/v5_manual/index.html). The five LAMP primer sets (two outer and two inner for each) designed by the software were experimentally tested for suitability. The screening of the appropriate primer set was performed and the optimal primer set (LM1) was selected for the downstream experiments. The selection of primers was based on criteria as described previously (33). The primer details are shown in Supplementary Table 1; Additional File 2. All primers were synthesized (and purified by HPLC) by Sangon Biotechnology Co., Ltd., Shanghai, China.

For comparison, two other primer sets (part of our unpublished results; Supplementary Table 2; Additional File 2) targeting 16s rDNA (*ssu rRNA* gene sequences of a number of microsporidian species; universal primer set) and *septin3* (*Nosema bombycis*) genes, respectively, were used in the primer screening experiment.

### Reaction system and reaction conditions

The DNA of *Nosema bombycis*, midgut of healthy silkworms, midgut of infected silkworms, and ddH_2_O were used as templates. The five LAMP primer sets as shown in Supplementary Table 1 were used to perform amplification at 63°C for 90 min. The amplification system (25 μL) comprised of: 12.5 μL 2 × Reaction Mix, 0.8 μmol/L each of inner primers (FIP and BIP), 0.2 μmol/L each of outer primers (F3 and B3), 8 U of Bst (*Bacillus stearothermophilus*) DNA polymerase, ddH2O and 2.0 μL of template DNA.

Where relevant, the reaction conditions were determined based on three methods: fluorescence amplification curve, agarose gel electrophoresis, and direct visual chromogenic (addition of SYBR green I fluorescent dye) methods. The amplification products were visually detected using 1.5% agarose gel electrophoresis. All reactions were performed in triplicates.

### Optimization of reaction temperature

1.2 × 10^−1^ ng/μl of *Nosema bombycis* spore DNA was used as a template. The optimal primer set (LM1) was used as the amplification primer. The LAMP amplification was performed at six different reaction temperatures: 60, 61, 62, 63, 64, and 65°C for 90 min.

### Optimization of concentration ratio of outer and inner primers

The primer concentration ratio was optimized using 1.2 × 10^−1^ ng/μL of *Nosema bombycis* spore DNA as a template. The LAMP amplification was performed in a constant temperature fluorescence detector using the optimal primer set (LM1) and amplification temperature. Details of reaction and amplification system are shown in Table 1. The reaction time was used to determine the optimal primer concentration ratio.

**Table 1:** LAMP amplification system for optimization of concentration ratio of outer and inner primers of *MetAP2* primer set (μL).

**Reaction system**	**Concentration ratio of outer primer to inner primer**			
	**1:4**	**1:6**	**1:8**	**1:10**
2 × Reaction mix	12.5	12.5	12.5	12.5
Bst DNA polymerase (8 U)	1.0	1.0	1.0	1.0
Fluorescent indicator (SYTO-9)	0.5	0.5	0.5	0.5
External primer F3 (10 μmol/L)	0.5	0.5	0.5	0.5
External primer B3 (10 μmol/L)	0.5	0.5	0.5	0.5
Inner primer BIP (40 μmol/L)	0.5	0.75	1	1.25
Inner primer FIP (40 μmol/L)	0.5	0.75	1	1.25
Template DNA	2	2	2	2
ddH_2_O	Up to 25			

### Specificity test

For performing specificity test, the DNA templates of *Nosema bombycis* spores, *Bacillus bombysepticus, Bacillus thuringiensis, Beauveria bassiana, BmCPV, BmNPV*, and *BmDNV* were used. Using the optimal primers (LM1), reaction temperature and primer ratio, a constant temperature fluorescence detector was used to perform the LAMP amplification and specificity testing. The reaction results were reflected by the amplification curve.

### Construction of MetAP2-pMD19T plasmid standard

The primer design strategy for construction of MetAP2-pMD19T plasmid standard cloning fragment was based on the outer primer amplification of the optimal primer set (LM1) of *MetAP2*, and linking of product to the pMD19T plasmid. The procedural details are given in Additional File 3.

### Sensitivity testing of plasmid standards

The concentration of the MetAP2-pMD19T plasmid standard following sequencing verification was measured using a UV spectrophotometer and converted into copy number. The specific calculation formula is as follows: plasmid copy number (copies/μL) = 10^−9^ × plasmid concentration (ng/μL) × 6.02 × 10^23^ (/mol)/660 × total length of recombinant plasmid (bp). Then 10-fold gradient dilution to 1.0 × 10^6^ copies/μL ~1.0 copies/μL. The LAMP amplification was carried out using a plasmid of 1.0 × 10^6^ copies/μL ~1.0 copies/μL as a template in a constant temperature fluorescence detector at 61°C for 90 min. The detection results were determined by gel electrophoresis and direct visual chromogenic methods.

### Practicality test

*Nosema bombycis* spore DNA (1.2 ng/μL, 1.2 × 10^−1^ ng/μL, 1.2 × 10^−2^ ng/μL, 1.2 × 10^−3^ ng/μL, 1.2 × 10^−4^ ng/μL, 1.2 × 10^−5^ ng/μL, 1.2 × 10^−6^ ng/μL) was used as a template. The optimal primer set was used as the amplification primer, and a constant-temperature fluorescence detector was used for the LAMP amplification.

### Therapeutic effect of fumagillin and application of LAMP in the detection of *Nosema bombycis* before and after fumagillin treatment

The collection and purification of *Nosema bombycis* spores was performed as described earlier in section Collection and purification of *Nosema bombycis*. Fumagillin was prepared into a medicinal solution with a concentration of 25 mg/L [manufacturer's recommended concentration for honeybee nosemosis (44)]. The silkworms were divided into eight treatment groups: 4A, 4B, 4C, 4D, 5A, 5B, 5C, and 5D. For methodological details, please see Additional File 3.

From each group, four silkworms were dissected and the midguts with the chyme removed (as much as possible) were stored in a −80°C refrigerator for later use. The dissected silkworms were observed with a 30 × stereomicroscope. Thereafter, the samples were taken every 6 h until silkworms become mature.

The DNA of *Nosema bombycis* spores, the midgut of silkworms infected with *Nosema bombycis* spores, the midgut of silkworms treated with fumagillin after infection with *Nosema bombycis* spores, and the midgut of healthy silkworms was extracted at different life stages. The LAMP amplification was performed using the LM1 primer set in a constant temperature fluorescence detector at 61°C for 90 min.

### Statistical analysis

One-way analysis of variance (ANOVA) was used to analyze data of body weight and cocoon quality indicators of 4th and 5th instar silkworms in different groups using SPSS software. Least significant difference was used as a *post hoc* test. Data are presented as the mean ± standard error of the mean (SEM). *P* < 0.05 was taken as significant. Where appropriate, all experiments were repeated at least three times and all LAMP reactions were performed in triplicates.

## Results

### Screening of LAMP primers

**Figure 1:**
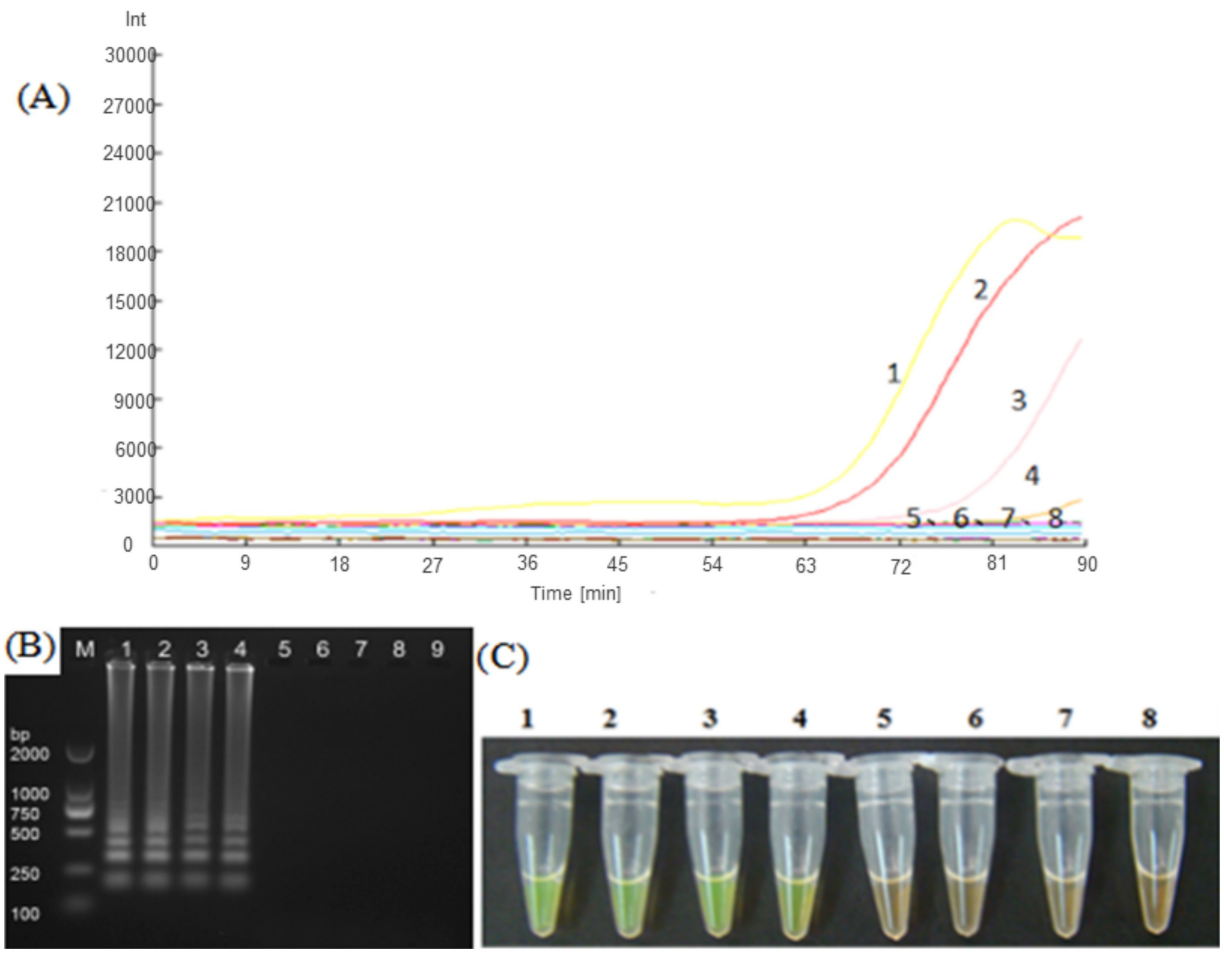
Screening of LAMP primers based on *MetAP2* target gene. **(A)** Represents the real-time monitoring of the fluorescence amplification−1: Universal primer, 2: *Septin3* primer, 3: LM1 primer, 4: LM5 primer, 5: LM2 primer, 6: LM3 primer, 7: LM4 primer, 8: Sterilized water. The vertical (y) axis represents relative fluorescence intensity and the horizontal (x) axis represents time in minutes. **(B)** Shows gel electrophoresis−1: Universal primer, 2: *Septin3* primers, 3: LM1 primer, 4: LM5 primer, 5: LM2 primer, 6: LM3 primer, 7: LM4 primer, 8: Sterilized water, 9: Blank. M: DL2000 Marker. **(C)** Shows visual (chromogenic) detection of LMAP amplicons after addition of SYBR green I fluorescent dye−1: Universal primer, 2: *Septin3* primer, 3: LM1 primer, 4: LM5 primer, 5: LM2 primer, 6: LM3 primer, 7: LM4 primer, 8: Sterilized water. After the LAMP reaction was completed, 1 μL of fluorescent dye (SYBR Green I) was added to the tube containing reaction mixture. After mixing with the reaction product, color development was observed with the naked eye under the natural light. Green color: DNA-dye complex; Orange/Brown color: No DNA-dye reaction.

The five LAMP primer sets (LM1–LM5) based on the *MetAP2* gene designed in the present study were screened for their ability to amplify the DNA template of *Nosema bombycis* spores. From these, the LM1 and LM5 primer sets were able to amplify the DNA template of *Nosema bombycis* (Figure 1). The primer sets LM1 and LM5 detected the fluorescence signals in around 50 min, with the LM1 primer set appearing earlier compared to the LM5, indicating its better detection specificity. The visual detection by chromogenic method was also consistent with the fluorescence amplification result (Figure 1). Hence, the LM1 primer set, based on the *MetAP2* target gene, was deemed suitable as the LAMP primer and used in the subsequent experiments.

### Optimization of reaction temperature

**Figure 2:**
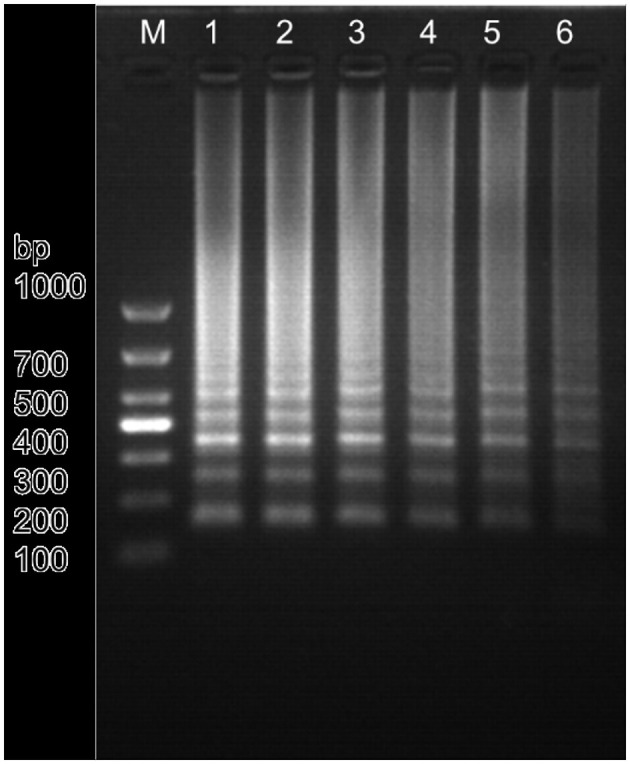
Amplification results of *Nosema bombycis*—positive plasmid template at different temperatures using the LM1 primer set. 1: 59.9°C. 2: 60.9°C. 3: 62.1°C. 4: 62.8°C. 5: 64.1°C. 6: 65.2°C. M: DL1000 Marker.

Based on the primer screening results, the LM1 primer set was used to amplify the positive plasmid template of *Nosema bombycis* spores at different amplification temperatures of 59.9, 60.9, 62.1, 62.8, 64.1, and 65.2°C. The results are shown in Figure 2. The amplification was detected with the LM1 primer set at different temperatures ranging from 60 to 65°C. As shown in Figure 2, typical ladder-shaped amplification bands were observed at different temperatures ranging from 60 to 65°C. The band produced at 60.9°C was deemed clear and brightest, and had the strongest fluorescence intensity (Figure 2; Lane 2). Therefore, 60.9°C was selected as the optimal amplification temperature for the LM1 primer set.

### Optimization of the concentration ratio of outer and inner primers

**Figure 3:**
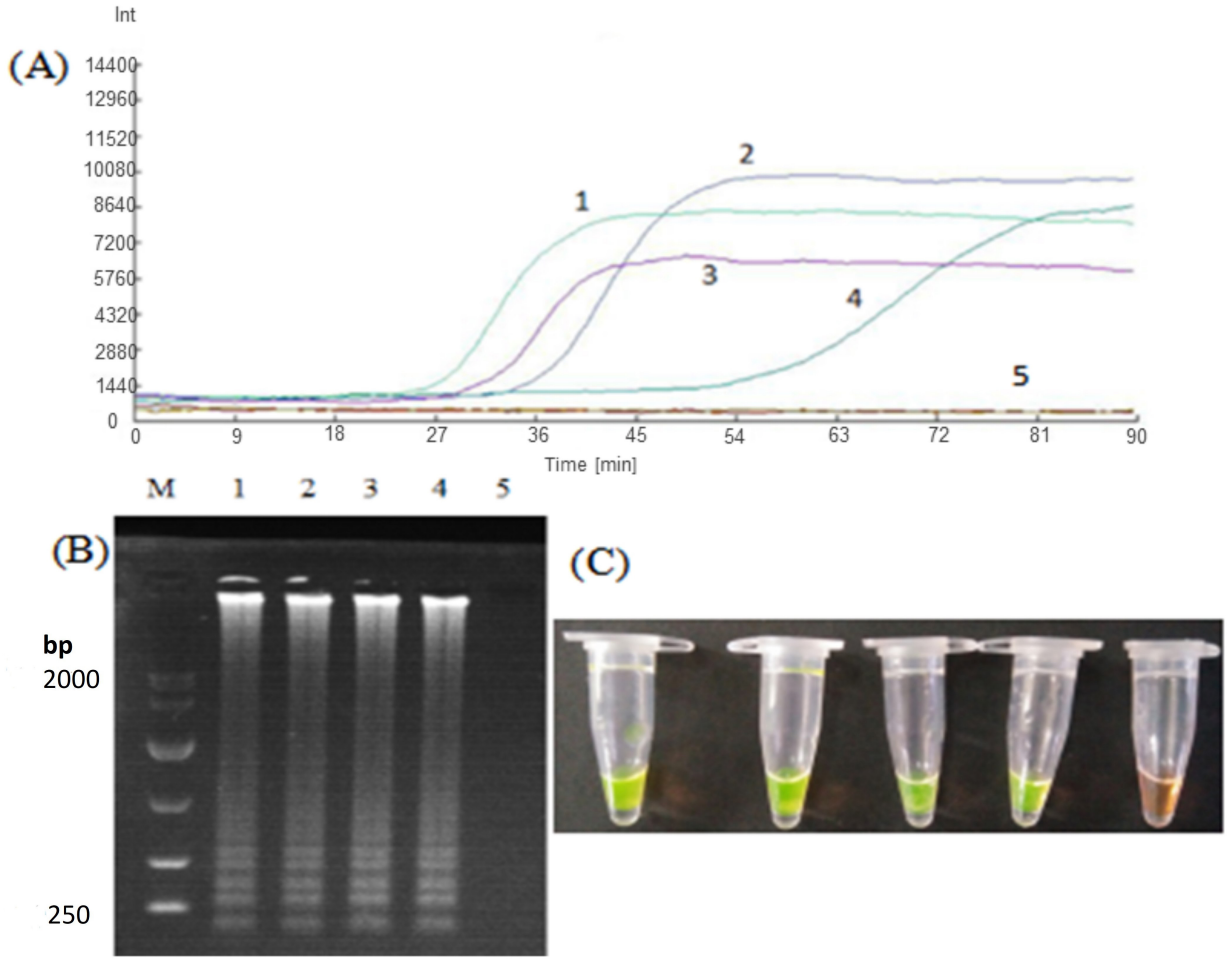
Optimization of outer to inner primer concentration ratio for LM1 primer set. **(A)** Represents the real-time monitoring of the fluorescence amplification for optimization of concentration ratio of the outer to inner primers for the LM1 primer set. 1: The outer primer: inner primer ratio 1:8. 2: outer primer: inner primer ratio 1:10. 3: outer primer: inner primer ratio 1:4. 4: outer primer: inner primer ratio 1:6. 5: Sterilized water. The vertical (y) axis represents relative fluorescence intensity and the horizontal (x) axis represents time in minutes. **(B)** Shows gel electrophoresis. 1: The outer primer: inner primer ratio 1:8. 2: outer primer: inner primer ratio 1:10. 3: outer primer: inner primer ratio 1:4. 4: outer primer: inner primer ratio 1:6. 5: Sterilized water. M: DL10000 Marker. **(C)** Shows visual (chromogenic) detection of LMAP amplicons after addition of SYBR green I fluorescent dye. 1: The outer primer: inner primer ratio 1:8. 2: outer primer: inner primer ratio 1:10. 3: outer primer: inner primer ratio 1:4. 4: outer primer: inner primer ratio 1:6. 5: Sterilized water. After the LAMP reaction was completed, 1 μL of fluorescent dye (SYBR Green I) was added to the tube containing reaction mixture. After mixing with the reaction product, color development was observed with the naked eye under the natural light. Green color: DNA-dye complex; Orange/Brown color: No DNA-dye reaction.

Using *Nosema bombycis* plasmid as a template at optimized amplification temperature, the ratios of outer and inner primers of the LM1 primer set were optimized as follows: outer primer: inner primer ratio of 1:8, outer primer: inner primer 1:10, outer primer: inner primer 1:4, outer primer: inner primer 1:6. The real-time fluorescence amplification, agarose gel electrophoresis and direct visual chromogenic results are shown in Figure 3. The results showed that when the ratio of outer primer: inner primer was 1:4, 1:6, 1:8, and 1:10, *Nosema bombycis* spore DNA template was detected. At the ratio of outer to inner primers 1:4, the amplification time was 34.5 min, and the relative fluorescence intensity was the highest. For 1:6, the amplification time was 52.5 min and the fluorescence intensity was relatively low. For 1:8, the amplification time was 26 min and the fluorescence intensity was higher. The amplification time of 1:10 was 29.5 min. As shown in Figure 3, the electrophoretic band at 1:8 primer ratio was brighter and deemed appropriate (Figure 3; Lane 1). Therefore, the ratio of outer primer to inner primer 1:8 having the shortest amplification time and the strongest fluorescence signal intensity was selected as the optimal primer concentration ratio of the LM1 primer set for subsequent experiments.

### Specificity verification

**Figure 4:**
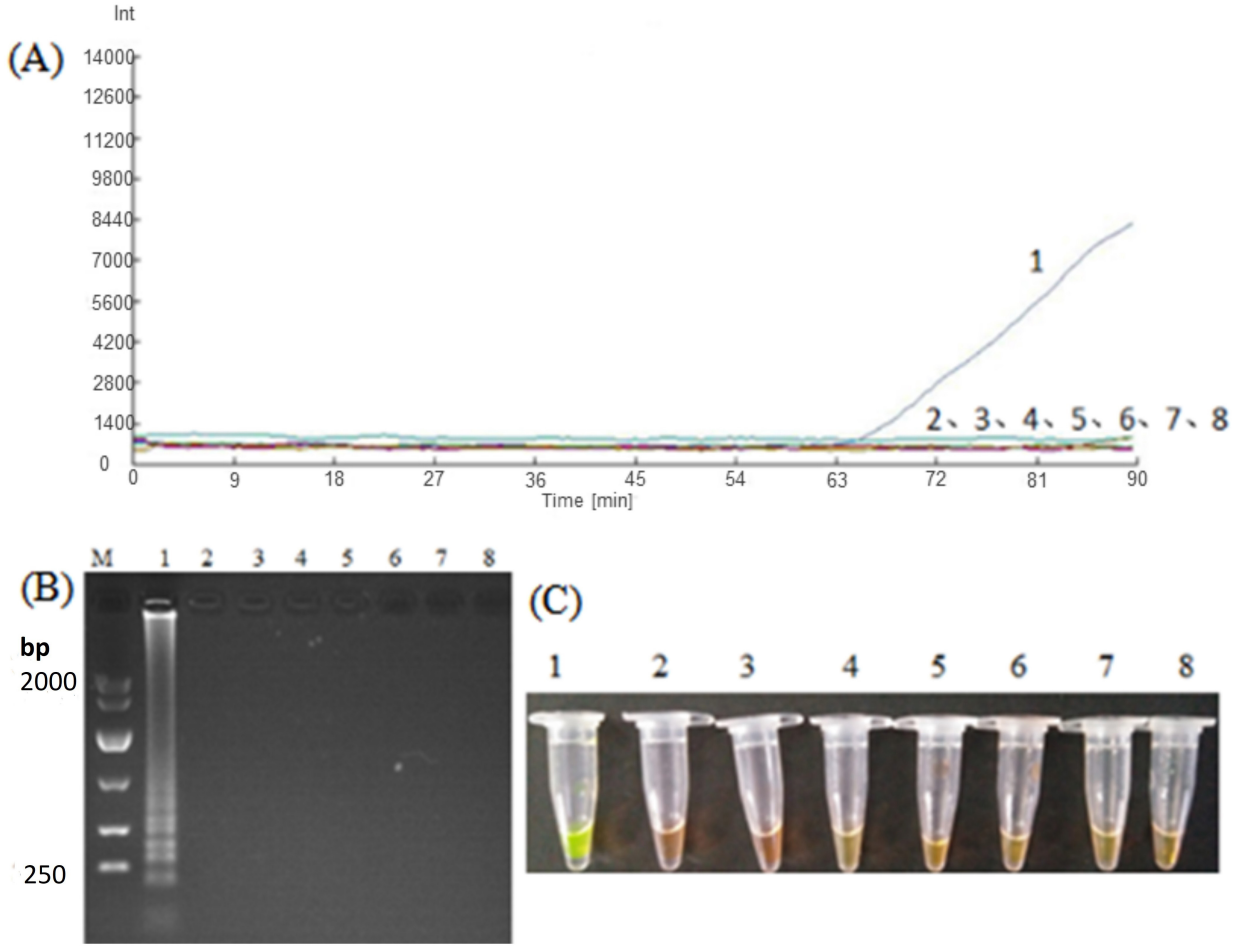
Amplification of *Nosema bombycis* and other microorganisms by LM1 primer set. **(A)** Represents the real-time monitoring of the fluorescence amplification. The vertical (y) axis represents relative fluorescence intensity and the horizontal (x) axis represents time in minutes. **(B)** Shows gel electrophoresis. **(C)** Shows visual (chromogenic) detection of LMAP amplicons. After the LAMP reaction was completed, 1 μL of fluorescent dye (SYBR Green I) was added to the tube containing reaction mixture. After mixing with the reaction product, color development was observed with the naked eye under the natural light. Green color: DNA-dye complex; Orange/Brown color: No DNA-dye reaction. 1: *Nosema bombycis*. 2: *Bacillus bombysepticus*. 3: *Bacillus thuringiensis*. 4: *Beauveria bassiana*. 5: *BmCPV*. 6: *BmNPV*. 7: *BmDNV*. 8: Sterilized water. M: DL10000 Marker.

For verification of specificity of LAMP, the DNA templates of *Nosema bombycis, Bacillus bombysepticus, Bacillus thuringiensis, Beauveria bassiana, BmCPV, BmNPV*, and *BmDNV* were used for amplification. As shown in Figure 4, it was observed that the LM1 primer set had fluorescent signals for the detection of *Nosema bombycis* at 63 min, and showed obvious ladder-type bands in the electrophoretic gel. Meanwhile no bands were observed for the DNA templates of other microorganisms and sterile water. This assay showed that the LM1 primer set can specifically amplify the DNA of *Nosema bombycis*.

### Sensitivity testing of LAMP

**Figure 5:**
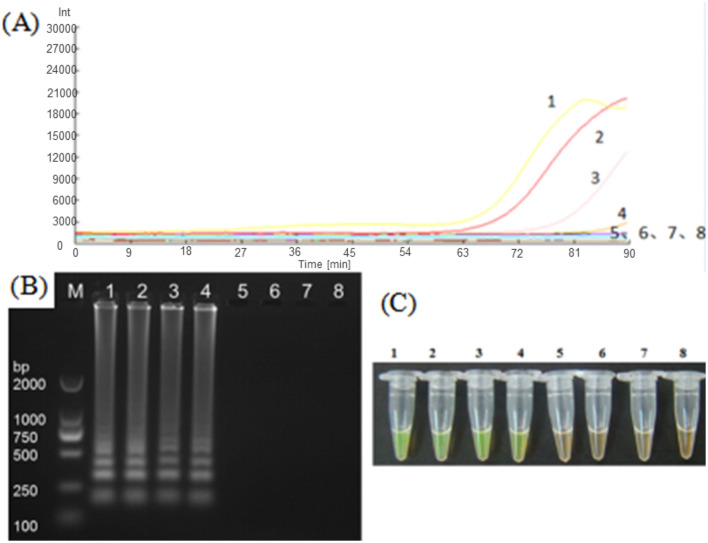
LAMP amplification of *Nosema bombycis* pMD-19T-met plasmid using LM1 primer set. **(A)** Shows the real-time fluorescence amplification. **(B)** Shows the results of agarose gel electrophoresis. **(C)** Shows visual (chromogenic) detection of LMAP amplicons. In **(A)**, The vertical (y) axis represents relative fluorescence intensity and the horizontal (x) axis represents time in minutes. In **(C)**, After the LAMP reaction was completed, 1 μL of fluorescent dye (SYBR Green I) was added to the tube containing reaction mixture. After mixing with the reaction product, color development was observed with the naked eye under the natural light. Green color: DNA-dye complex; Orange/Brown color: No DNA-dye reaction. 1: 10^6^ copies of *Nosema bombycis* -positive plasmid. 2: 10^5^ copies of *Nosema bombycis* -positive plasmid. 3: 10^4^ copies of *Nosema bombycis* -positive plasmid. 4: 10^3^ copies of *Nosema bombycis* -positive plasmid. 5: 10^2^ copies of *Nosema bombycis* -positive plasmid. 6: 10^1^ copies of *Nosema bombycis* -positive plasmid. 7: 10^0^ copies of *Nosema bombycis* -positive plasmid. 8: Sterilized water. M: DL2000 Marker.

In order to verify the sensitivity of the LAMP to the plasmid standard pMD-19T-met, plasmids with different dilution concentrations were used as templates and amplified with the LM1 primer set for detection. As shown in Figure 5, although the detection time was relatively longer (~80 min), LMAP could detect the *Nosema bombycis* pMD-19T-met positive plasmid even at the lowest concentration of 10^3^ copies. Obvious bands were also observed in the electrophoretic gels, with greater brightness of the band observed at the higher plasmid concentration. The results of fluorescence amplification, agarose gel electrophoresis and direct visual chromogenic assays were consistent, indicating that the LAMP can specifically amplify the plasmid standard pMD-19T-met, and was sensitive even at the lower concentration of the plasmids.

### Practical testing of LAMP

**Figure 6:**
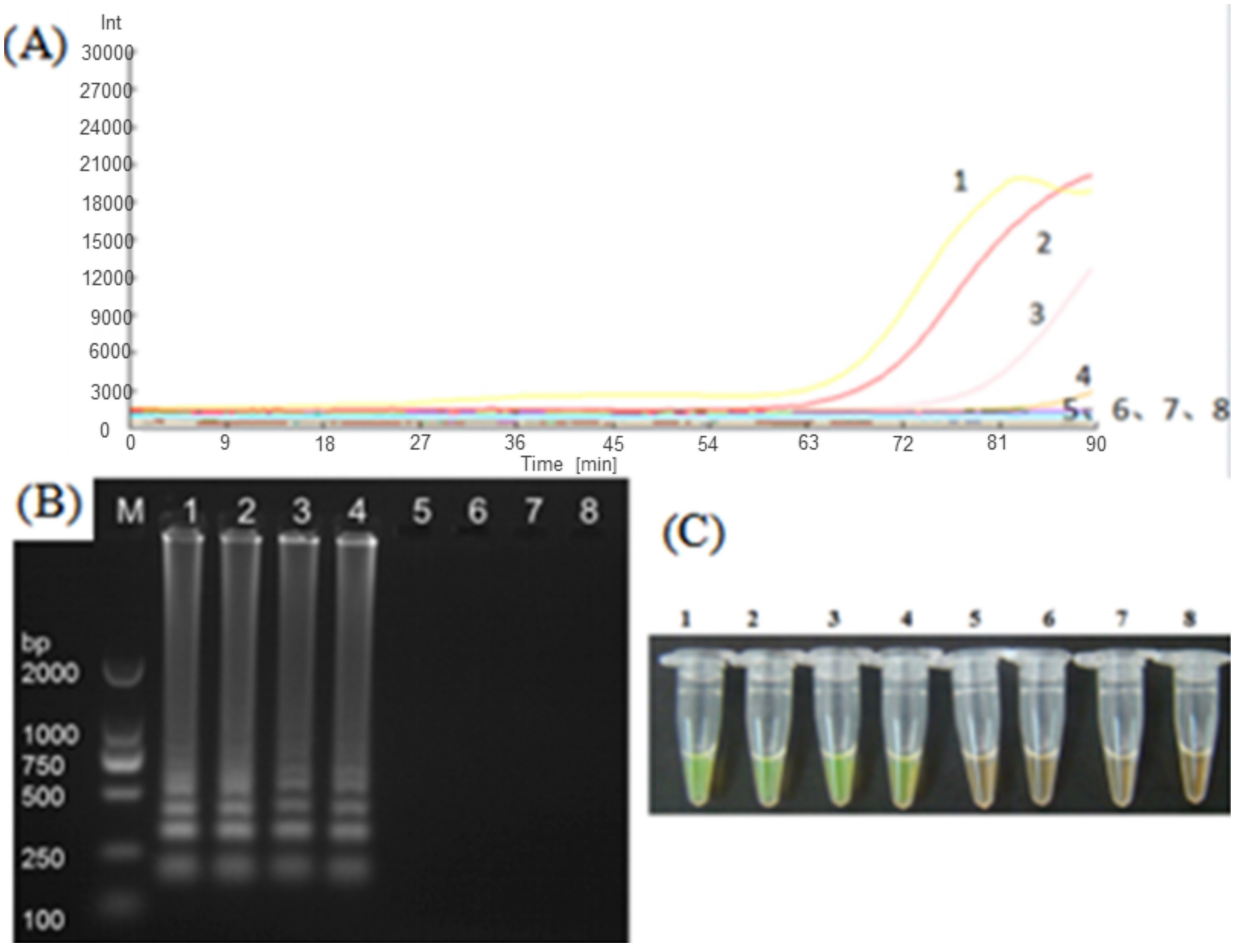
LAMP amplification of *Nosema bombycis* DNA using LM1 primer set. **(A)** Shows the real-time fluorescence amplification. **(B)** Shows the results of agarose gel electrophoresis. **(C)** Shows visual (chromogenic) detection of LMAP amplicons. In **(A)**, The vertical (y) axis represents relative fluorescence intensity and the horizontal (x) axis represents time in minutes. In **(C)**, After the LAMP reaction was completed, 1 μL of fluorescent dye (SYBR Green I) was added to the tube containing reaction mixture. After mixing with the reaction product, color development was observed with the naked eye under the natural light. Green color: DNA-dye complex; Orange/Brown color: No DNA-dye reaction. 1: *Nosema bombycis* DNA 10^0^ ng/μL. 2: *Nosema bombycis* DNA 10^−1^ ng/μL. 3: *Nosema bombycis* DNA 10^−2^ ng/μL. 4: *Nosema bombycis* DNA 10^−3^ ng/μL. 5: *Nosema bombycis* DNA 10^−4^ ng/μL. 6: *Nosema bombycis* DNA 10^−5^ ng/μL. 7: *Nosema bombycis* DNA 10^−6^ ng/μL. 8: Sterilized water. M: DL2000 Marker.

In order to verify the practicality of the LAMP assay, different dilution concentrations of *Nosema bombycis* spore DNA were used as templates for amplification using the LM1 primer set. As depicted in Figure 6, the detection time of the LM1 primer set was relatively longer. At DNA concentration of 1.2 ng/μL, the real-time fluorescence signal appeared in ~60 min. The lowest concentration of *Nosema bombycis* spore DNA detected was 10^−3^ ng/μL. Obvious bands were observed in electrophoretic gels, with greater brightness of band observed at the higher the concentration, indicating that the LM1 primer set can specifically amplify the *Nosema bombycis* spore DNA with good sensitivity.

### Observation results of fumagillin treatment against *Nosema bombycis* infection in silkworms

The representative photographs showing the appearance of adults, pupae, and cocoons in different groups are shown in Supplementary Figure 1 (Additional File 4). The adults, pupae, and cocoons of silkworms treated with fumagillin were apparently larger compared to those without fumagillin. In Supplementary Figures 1A, B, typical lesions of silkworm pebrine can be seen. In Supplementary Figure 1C, it can be seen that the cocoon shells of the *Nosema bombycis*-infected silkworms were thinner and more transparent, compared to those treated with fumagillin following infection.

The morphological (stereomicroscopic observation) features of body parts of silkworms in healthy, infected, and infected + fumagillin-treated groups are shown in Supplementary Figure 2 (Additional File 4). It can be seen that the heads of the healthy and infected + fumagillin-treated silkworms were whiter, with relatively complete and clean legs and spiracles (Supplementary Figures 2A2, B2, C2, A3, B3, and C3). The infected silkworms were significantly darker in color due to black spots on their entire bodies. The thorax, abdominal, and caudal legs were all black and partially ulcerated (Supplementary Figures 2A1, B1, C1), potentially due to the excessive spore count in the later stages of infection.

As shown in Supplementary Figure 3 (Additional File 4), the midguts of healthy (Supplementary Figures 3A1–A3) and infected + fumagillin-treated (Supplementary Figures 3C1–C3) silkworms had normal yellowish color, with transparent silk glands. While infected silkworms (Supplementary Figures 3B1–B3) exhibited white patches in their midguts and silk glands with opaque appearance. This result indicated that fumagillin had a certain therapeutic effect against *Nosema bombycis* infection in silkworms.

### Effects of fumagillin on silkworm indicators

The body weight and cocoon quality indicators in different groups of both 4th instar (4A, B, C, and D) and 5th instar (5A, B, C, and D) life stages of silkworms are shown in Table 2. The body weights of silkworms between different groups showed significant differences, with silkworms infected (non-treated) with *Nosema bombycis* spores returning lower parameters compared to other three groups in both 4th and 5th instar life stages. Similarly, the cocoon quality indicators were relatively lower in silkworms infected with *Nosema bombycis* spores. Of note, silkworms that were infected and then treated with fumagillin showed comparable body weights and cocoon quality indicators with healthy (blank control) and fumagillin only groups.

**Table 2:** Body weight and cocoon quality indicators of 4th and 5th instar silkworms in different groups (mean + SEM).

**Life stage**	**Experimental groups**	**Body weight**	**Cocoon quality index**	
			**Amount of cocoon layer**	**Amount of whole cocoon**
Fourth instar (258 h after treatment)	4A	2.15 ± 0.01^c^	0.24 ± 0.03^b^	1.09 ± 0.05^ab^
	4B	2.23 ± 0.04^b^	0.28 ± 0.01^a^	1.20 ± 0.10^a^
	4C	2.29 ± 0.01^a^	0.23 ± 0.01^b^	1.08 ± 0.03^b^
	4D	2.01 ± 0.04^d^	0.26 ± 0.01^ab^	1.16 ± 0.04^ab^
Fifth instar (138 h after treatment)	5A	2.20 ± 0.02^b^	0.25 ± 0.01^a^	1.15 ± 0.01^a^
	5B	2.37 ± 0.03^a^	0.25 ± 0.02^a^	1.19 ± 0.01^a^
	5C	2.23 ± 0.07^b^	0.23 ± 0.03^a^	1.10 ± 0.11^a^
	5D	2.18 ± 0.07^b^	0.18 ± 0.02^b^	0.98 ± 0.02^b^

*n* = 50 in each group. Values are shown in grams. Values with different superscripts in the same column are different at each life stage. Fourth instar life stage−4A: healthy silkworms (blank control), 4B: Fumagillin only, 4C: infected with *Nosema bombycis* spores and then treated with fumagillin, 4D: infected with *Nosema bombycis* spores only. Fifth instar life stage−5A: healthy silkworms (blank control), 5B: Fumagillin only, 5C: infected with *Nosema bombycis* spores and then treated with fumagillin, 5D: infected with *Nosema bombycis* spores only.

### Application of LAMP in the detection of *Nosema bombycis* following fumagillin treatment

**Figure 7:**
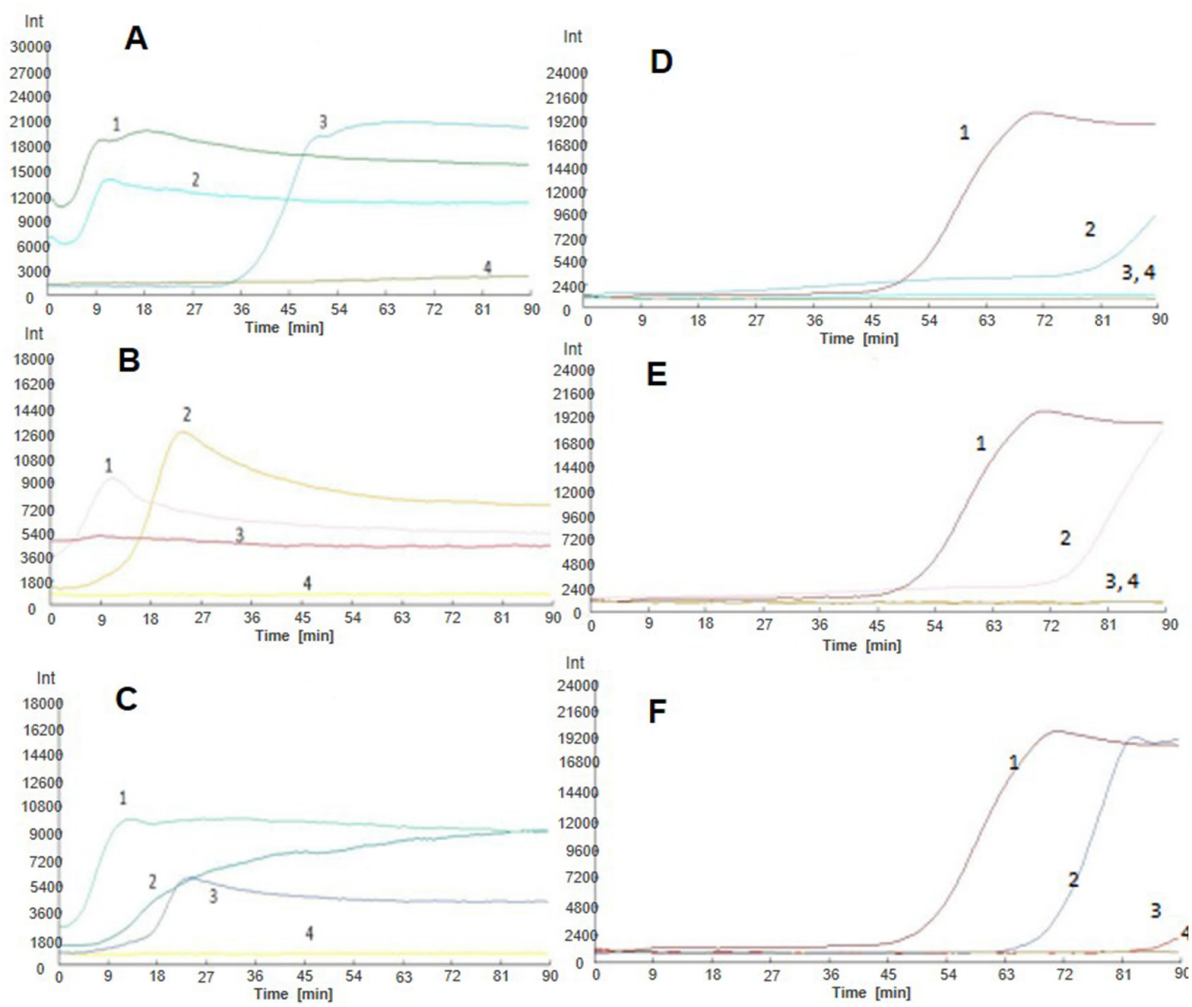
LAMP-based detection of *Nosema bombycis* in silkworms at different life stages before and after fumagillin treatment. **(A–F)** Show real-time fluorescence amplification at 6, 18, 42, 90, 138, and 150 h post-fumagillin treatment. Vertical (y) axis represents relative fluorescence intensity and the horizontal (x) axis represents time in minutes. 1: DNA of pure *Nosema bombyci*s spores. 2: DNA of the midgut of silkworms infected with *Nosema bombycis* spores. 3: DNA of the midgut of silkworms infected with *Nosema bombycis* spores and then treated with fumagillin. 4: Sterilized water.

In order to practically test the effectiveness of the developed LAMP assay, we carried out real-time fluorescence amplification using the LM1 primer set at the optimized reaction conditions to amplify the DNA templates of *Nosema bombycis* spores, the midgut of silkworms infected with *Nosema bombycis* spores, the midgut of silkworms treated with fumagillin after infection with *Nosema bombycis* spores, and the midgut of healthy silkworms. The amplification results are shown in Figure 7. Briefly, the lack of amplification in the control (sterilized water) indicated that the reaction system was free of contamination. Overall, the amplification of LAMP showed better results in terms of detection, with shorter overall amplification time. However, the relative fluorescence intensity was lower. Importantly, the DNA of the pure *Nosema bombycis* spores, the midguts of infected silkworms, and the midguts of infected silkworms treated with fumagillin showed amplification at early stages (7A–C). Meanwhile, the DNA of the pure *Nosema bombycis* spores and the midguts of silkworms infected with *Nosema bombycis* spores showed amplification throughout all life stages, indicating the diagnostic capability of the LAMP assay to detect the infection. From these, the amplification of DNA of pure *Nosema bombycis* spores appeared earlier and had stronger fluorescence values. The amplification time of DNA of the midgut of silkworms infected with *Nosema bombycis* was also relatively early, compared to those treated with fumagillin following infection. In this case, the fluorescence intensity was stronger at the early stages of infection and the amplification time in the later stages was relatively longer than the DNA template of pure *Nosema bombycis* spores. In particular, at 90 h and later, no real-time fluorescence amplification signals were recorded for the DNA of the midguts of silkworms infected with *Nosema bombycis* spores and then treated with fumagillin.

## Discussion

As a devastating microsporidian disease of silkworms, pebrine is the only disease with a mandatory quarantine (4) that causes a huge economic losses to the sericulture industry. The causal agent of pebrine, *Nosema bombycis*, is a mysterious pathogen with complex biology and high pathogenicity (42). At present, the availability of well-established molecular diagnostic tools with practical field application is limited. Although, attempts have been made in the past to identify possible pharmacological targets and to develop treatment/therapeutic strategies to cure microsporidiosis, unfortunately the results of those studies have ranged from promising to disappointing [reviewed in (14)]. In particular, the drugs like albendazole and fumagillin have been reported to be effective against some of the human and insect (e.g., honeybee) microsporidia, but reporting on the use of these drugs in managing pebrine in silkworms is almost non-existent. At the field level, chemical and physical methods like application of disinfectants such as organic and inorganic chlorines and formaldehyde are adopted in an attempt to prevent, or at least minimize, the risk of pebrine infection in silkworm rearing houses (49). Unfortunately, these and other similar methods come with limited effectiveness, providing little or no relief to the farmers (personal communication). It is, therefore, highly desired that new logical and effective approaches for the prevention and control of pebrine infections in silkworms should be tested.

Previously, it was argued that essential protein coding genes of *Nosema bombycis* should be tested as logical cellular targets for developing therapeutic (42) and diagnostic (26) strategies for this deadly microsporidian parasite of wild and domestic silkworms. The good news is that, in recent times, a few molecular diagnostic assays have been developed to facilitate the diagnosis of pebrine infection in silkworms. But currently there is a lack of studies in which essential protein coding genes of *Nosema bombycis* have been exploited as a target for the development of field-applicable diagnostic assay. Therefore, in the present study, we aimed to establish a specific LAMP assay based on *MetAp2* as a target gene. Given that *MetAp2* is a known cellular target of fumagillin, we also evaluated the potential efficacy of fumagillin against *Nosema bombycis* infection in domestic silkworms. To the best of our knowledge, this is the first report in which a specific and field-applicable LAMP assay is established targeting a gene (*MetAp2*) that is not only an essential protein coding gene, but also a logical therapeutic target of a pharmacological (fumagillin) drug against *Nosema bombycis*.

In recent times, the LAMP assay has been regarded as a feasible DNA-based alternative molecular assay for sensitive and specific detection of infectious pathogens (50). A number of previous studies have reported usefulness and field-friendliness of LAMP in rapid and efficient detection of infectious pathogens of economic significance in a wide range of animal species [reviewed in (51)]. In terms of limit of detection (LoD) and amplification time, the LAMP has been reported to produce better results compared to conventional PCR, nested PCR and real-time PCR techniques (50). Primer design and concentration have a bearing on efficiency and specificity of LAMP amplification (50, 52, 53). In the present study, LM1 and LM5 primer sets were able to produce fluorescence signals in around 50 min, but the LM1 primer set produced better results, and hence it was used in the downstream analysis. The reaction temperature of 60.9 C was found to be optimal. Meanwhile, outer primer to inner primer ratio of 1:8 was found to be optimal, with the shortest amplification time and the strongest fluorescence intensity. The established LAMP assay showed high specificity for the DNA of *Nosema bombycis* spores, as the templates of other microorganisms including *Bacillus bombysepticus, Bacillus thuringiensis, Beauveria bassiana, BmCPV, BmNPV*, and *BmDNV* showed no amplification. Previously, it was shown that the outer primer to inner primer ratio of 1:8 produced the strongest amplification when LAMP was used to detect *Brucella* spp. in raw milk and blood of sheep (54), and *Clostridium piliforme* in infected mice (55). Similarly, the LAMP assay showed high specificity for *Brucella abortus* and didn't amplify non-*Brucella* spp. (54). Similar findings were also reported when LAMP was applied for the detection of *Clostridium piliforme* in experimentally infected mice (55), *Vibrio vulnificus* in raw oysters (56), pox viruses in sheep and goats (57). In the present study, the LAMP assay was able to detect pMD-19T-met positive plasmid at the lowest concentration of 10^3^ copies, with a detection time of ~80 min. The practicality test showed that the LAMP assay could detect *Nosema bombycis* spore DNA at the lowest concentration of 10^−3^ ng/μL. At concentration of 1.2 ng/μL, the real-time fluorescence signals appeared in ~60 min. The effectiveness of the developed LAMP assay was practically tested for the detection of *Nosema bombycis* in artificially infected silkworms treated with and without fumagillin at different life stages. The LAMP was able to detect *Nosema bombycis* at all life stages of untreated silkworms. Similarly, in fumagillin treated silkworms, no real-time fluorescence amplification signals were observed at 90 h and later, indicating the reliability of the LAMP in detecting *Nosema bombycis* DNA, and effectiveness of fumagillin in treating, at least to a certain degree, pebrine infection.

Previously ours and a few other research groups have developed the LAMP assays based on different target genes for the detection of pebrine in silkworms. In our previous study (35), *EB1* gene of *Nosema bombycis* (Guangdong isolate) was targeted to develop a LAMP assay to detect (at concentration of 5.0 × 10^−3^ ng/μL) pebrine infection in silkworms. Importantly, the scope of that LAMP assay (35) was different, as we aimed to detect *Nosema bombycis* in the silkworm eggs and not at different life stages of silkworms, as was the case in the present study. Therefore, in that case, the selection of *EB1* gene was rationale, because this gene is potentially related to the replication/maturation of spores of *Nosema bombycis*. Esvaran et al. (36) reported a LAMP assay targeting the *polar tube protein 1* (*PTP1*) gene of *Nosema bombycis* (Indian isolate). This gene is found in the polar tube [a unique invasion apparatus (58)] of microsporidian parasites and its expression/activity is believed to be linked with the appropriate environmental stimulation. For instance, it was reported that the polar tube can evert rapidly out of the microsporidian spore, forming a hollow tube which serves as a conduit for the passage of infectious cargo (sporoplasm and nuclear material) into a new host cell (58, 59). Based on this evidence, it can be assumed that the *PTP1* gene, like *EB1*, might be more suitable for the detection of *Nosema bombycis* parasites in the silkworm eggs, because it is where the sporoplasm is injected and undergo maturation (8). However, given the lack of concrete evidence on these aspects, we believe that it would be helpful to conduct focused work on this agenda in the future studies.

The other three papers (37–39) report LAMP assays based on the *small subunit ribosomal RNA* (16S *rRNA*) gene, for which enough concerns have been raised recently (27, 40, 41). Previously, it has been shown that the organization of *rRNA* gene of *Nosema bombycis* is very different and is the reverse of the organizational sequence found in the other previously known sequences of microsporidian *rRNAs* (60). In addition, it was argued that it is indeed hard to completely sequence *rRNAs* of microsporidian spp. due to inherent difficulty in designing suitable primer sets when the microsporidian *rRNA* sequences are highly diverse (60). In fact, it was shown that some isolates of *Nosema bombycis* also possess fragmented copies of *rRNA* genes (61, 62). Given that the *rRNA* repeat unit exists in multiple copies with intragenomic variation in gene order, integrity and sequence, it is a potential source of confusion in phylogenies based entirely upon 16S *rRNA* gene sequences (62). This evidence led to an argument that *rRNA* sequences are unreliable, even in the phylogenetic studies on microsporidian spp. (e.g., genus *Nosema*/*Vairimorpha*; (62)). In support of this, there have been some reports of inconsistent amplification in PCR-based assays due to the presence of multiple copies of 16S *rRNA* genes in *Nosema* genome (41, 63–65). This evidence led to a notion that other stable and single-copy genes should be preferred for developing molecular diagnostic assays for the detection of microsporidian spp., including *Nosema*. Interestingly, in a previous study (40), a LAMP assay based on *SSU rRNA* nucleotide sequence of *Nosema bombi* (microsporidium of bumblebees) non-specifically detected the DNA of *Nosema ceranae* (microsporidium infecting both honey bees and bumblebees). These LAMP primers also resulted in unreproducible amplification at some instances, leading to a recommendation from the authors that a specific primer set targeting other genes should be tested to eliminate these issues (40). Above evidence provides enough justification to rule out the selection of *rRNA* gene sequences for the development of diagnostic assays for *Nosema* spp., including *Nosema bombycis*. In addition to above limitations, the LAMP assay (based on *LSU rRNA* and *SSU rRNA* sequences) developed by Yan et al. (38) involved nucleic acid extraction through treatment with acid-washed glass beads and FTA cards. Although this assay was efficient and sensitive (3 × 10^4^ spores/mL) in detection of *Nosema bombycis*, but it came with an element of inconvenience related to the use of glass beads and FTA cards, and hence can't be considered as a cost-effective and field-friendly option.

In addition to LAMP assays discussed above, several reports of molecular diagnostic assays based on more advanced techniques requiring sophisticated equipment and higher technical skill sets are available in the literature. A real-time quantitative PCR assay based on the *small-subunit rRNA* gene was reported to detect *Nosema bombycis* in single silkworm eggs and newly hatched larvae with high sensitivity (0.1 spore DNA; 4). But the detection time of this assay was over 2.5 h (4). In order to avoid issues related to *rRNA* gene, a real-time quantitative PCR assay targeting an essential gene β*-tubulin* was developed to detect *Nosema bombycis* in different infected tissues of silkworms (27). This assay was able to detect 100 pg/μL of *Nosema bombycis* spores (27). More recently, a highly sensitive TaqMan assay was developed to detect *Nosema bombycis* in silkworms (16). This assay was capable of detecting as few as 10^2^ copies of the target (β*-tubulin*) gene. In addition, a newer amplification-free/CRISPR-Cas12a assay was recently reported to have high specificity and sensitivity (as low as 2 pg of genomic DNA) for the detection of *Nosema bombycis* in silkworms (30). Similarly, CRISPR/Cas12a fluorescence and CRISPR/Cas12a immunochromatographic detection methods were shown to have high specificity and sensitivity (2 fg/μL; (66)). Importantly, although all methods discussed above have shown good sensitivity and rapidity, but currently they seem to be far from having a practical application in the field, as they require sophisticated infrastructure, higher technical skills, and cost of probes compared to the LAMP assay. For instance, CRISPR/Cas12a fluorescence integrated with the Recombinase Polymerase Amplification (RPA) technology was indeed sensitive in the detection of *Nosema bombycis* (66), but it has certain limitations. Importantly, availability of standard RPA reagents, expensive kits, complex primer design, and laborious optimization of amplification system are some of the drawbacks that have limited its application to laboratory research so far (67, 68). Other nucleic acid-based diagnostic tools such as fluorescent *in situ* hybridization (FISH) have also been used for the detection of human microsporidia; however, FISH is less sensitive than PCR due to the lack of signal amplification (69, 70). In addition, the use of serological (immunodiagnostic) techniques has been reported for the detection of human microsporidia. But serology is not considered as a good molecular diagnostic tool (a least for the insect microsporidia spp.), and is more suitable for laboratory research (70).

In conventional microscopic inspection, the earliest possible detection of *Nosema bombycis* is around 72 h, which is based on the observation of pear-shaped sporont in silkworms (38). The LAMP assay developed in the present study was able to detect *Nosema bombycis* in infected silkworms as early as 30 and 42 h post-infection. In conformity to our finding, previous reports have also shown that the LAMP assay could detect *Nosema bombycis* 48 h post-infection, which is 24 h earlier compared to the routine microscopic inspection performed at the silkworm seed production centers (37, 38).

Fumagillin, the fermentation product of *Aspergillus fumigatus*, is currently the only approved veterinary drug to treat nosemosis in honeybees (44, 46, 71, 72). It covalently binds to *MetAp2* of microsporidia, causing irreversible inhibition by interfering with protein homeostasis and posttranslational modification essential for normal cellular function (43, 70, 73, 74). Homology modeling-based studies have shown that fumagillin covalently binds to His_231_ residue in human MetAP2 that is also highly conserved in MetAP2 sequences of microsporidia (70, 75, 76). It was reported that microsporidian *MetAP2* gene doesn't have any closer relationship to that of the other eukaryotes (43, 74), making it a highly logical therapeutic target against microsporidiosis. The characterization of the *MetAP2* gene of honeybee *Nosema* spp. has shown that, although *Nosema* spp. responded differently to fumagillin treatment, no apparent differences were observed in fumagillin binding sites in their sequences (44). It was further shown that *MetAP2* gene sequences of *Nosema apis, Nosema bombi*, and *Nosema ceranae* differed from those of humans and honeybees at two fumagillin binding sites (44). Previously, fumagillin was shown to be effective against a number of parasitic pathogens [discussed in (77)], including human [*Enterocytozoon bieneusi, Encephalitozoon intestinalis and Vittaforma corneae*; (70, 78)], honeybee [*Nosema cerenae* and *Nosema apis*; (44, 71, 72)], and fish (*Pleistophora anguillarurn*; (79)) microsporidia. Fumagillin had over 70% inhibition (*in vitro*) rate against protozoan parasite *Trichomonas gallinae* (77). This evidence and the promising characteristics of fumagillin highlight its potential to be tested as a therapeutic agent against *Nosema bombycis* infection in silkworms. In the present study, both 4th and 5th instar silkworms artificially infected with *Nosema bombycis* spores and then treated with fumagillin showed better body weight and cocoon quality indicators compared to those without fumagillin treatment. Tellingly, at 90 h and later, the LAMP assay showed no amplification of *Nosema bombycis* DNA in the midguts of infected silkworms treated with fumagillin. This indicated that continuous fumagillin treatment throughout all life stages of silkworms had certain inhibitory effect against *Nosema bombycis* infection. There is only one previous study (80) reporting the effect of fumagillin treatment against *Nosema bombycis* (Indian isolate) infection in domestic silkworms. It was reported that fumagillin at a concentration of 20 mg/mL (fed orally on alternative days) for 120 h of post-infection was able to reduce spore load and multiplication of *Nosema bombycis* (80). Although that study showed similar positive effect of fumagillin as reported in the present study, there were obvious differences in methodology and application of fumagillin. For instance, in that previous study, fumagillin was administrated after 12 h of post-infection, at a higher dose (20 mg/mL and above), with an interval of 24 h between two doses, and fed only up to 120 h of post-infection. On the contrary, in the present study, the concentration of fumagillin used was lesser (25 mg/L) and it was fed once daily from 6 h post-infection and continuously up to 258 h (fourth instar) and 138 h (fifth instar). Tellingly, the recommended concentration (25 mg/L) of fumagillin for treatment of honeybee nosemosis was reported to suppress reproduction of *Nosema ceranae* and *Nosema apis*, resulting in a very low count of spores in midgut tissues and hindgut content (44). Based on the results of the treatment of honeybee nosemosis under field conditions, it was shown that certain factors such as the length of time, temperature, and medium in which fumagillin application was made, negatively impacted the stability and concentration of the active ingredient (44, 71). Given the complexities of the silkworm rearing systems, it remains to be seen if fumagillin use has any practical value in the sericulture industry. In any case, given the limited evidence on the effectiveness of fumagillin against *Nosema bombycis*, it would be necessary to further validate the finding of the present study and that of others (80) by conducting more focused laboratory research and also through comprehensive field trials.

## Conclusion

**Figure 8:**
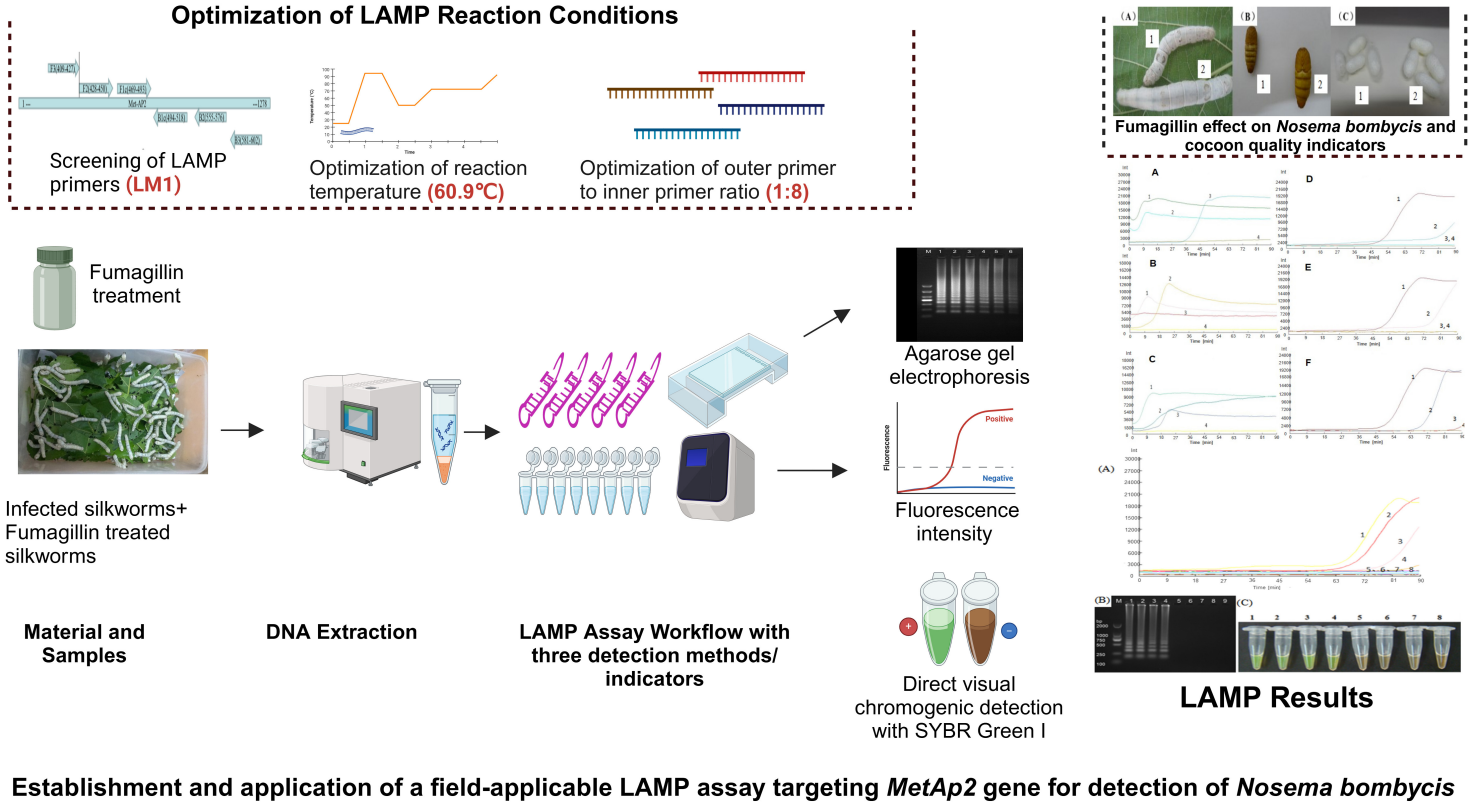
Graphical summary. Prepared using BioRender software (https://BioRender.com).

In the present study, a highly specific, sensitive, and field-applicable LAMP assay targeting *MetAP2* gene was developed for the detection of *Nosema bombycis* infection in silkworms. Meanwhile, fumagillin treatment (pilot trial) showed a certain degree of effectiveness in limiting *Nosema bombycis* infection in silkworms. The graphical summary of the developed LAMP assay and fumagillin effect is presented in Figure 8.

Overall, this study holds good promise for its practical application in the detection, control, and treatment of pebrine in the field settings. However, we propose that adequately powered field trials should be conducted to further validate the fumagillin effect against the pebrine disease in silkworms.

## Data Availability

Publicly available datasets were analyzed in this study. This data can be found here: Genbank, accession number: KX185053.1.

## Footnotes

^1^ https://einsteinmed.edu/departments/pathology/research/microsporidia/
