# Supplementary material for: Establishment and application of a loop-mediated isothermal amplification method based on MetAP2 gene for the detection of Nosema bombycis in silkworms (Bombyx mori)
Source: Front Vet Sci. 2025 Mar 10;12:1549224. doi: 10.3389/fvets.2025.1549224 (PMC11931649; doi:10.3389/fvets.2025.1549224)
Supplement: Supplementary file 1 [file Table_1.docx]

**Establishment and Application of a Loop-Mediated Isothermal Amplification Method Based on *MetAP2* Gene for the Detection of *Nosema bombycis* in Silkworms (*Bombyx mori*)**

Izhar Hyder Qazi^1*^, Ting Yuan^1^, Sijia Yang^1^, Christiana Angel^2, 3^, Jiping Liu^1^*

^1^Guangdong Provincial Key Lab of Agro-Animal Genomics and Molecular Breeding, College of Animal Science, South China Agricultural University, Guangzhou 510642, Guangdong, China

^2^Key Laboratory for Agro-Ecological Processes in Subtropical Region, Institute of Subtropical Agriculture, The Chinese Academy of Sciences, Changsha, China; the University of Chinese Academy of Sciences, Beijing, China

^3^Shaheed Benazir Bhutto University of Veterinary and Animal Sciences, Sakrand, Pakistan

*Corresponding authors: IHQ (vetdr_izhar@yahoo.com); JL (liujiping@scau.edu.cn)

**Additional file 1: Full length sequence of *MetAp2* of *Nosema bombycis***

ACCACGTCATGTTTTTTAATCTTTTTGTATTTTTTTTCTGCTCCCCTAAATGAGGCCTATTGTTTTATCAGAAGTCGAAGAAAAACCAATAGAATTTTTAGAAAAAGACGAGAAATACATAAAAAACGTCTTTTATGACAAAAACAATAATGAAATACCCAATGAACTTGAAAATGACATTCTTTTAGAAGCGAGGCGTGCAGCTGAAGCGCATAGAAGAATAAGATATAAAGTACAAAATTTAATTAAACCCGGGATACCTATAATAGATATCGTTAATTGTATAGAAAATTCAACAAGAACTCTTTTAAAGGGGGAGAAGGGTGATGGAATAGGGTTCCCTGCCGGGATGAGTGCTAATGATTGTGCTGCTCATTTTACCGTGCTTCCAGACGATAACACTACGACTTTACAAGAAAATGATGTATTAAAAATAGATTTTGGGACACATGTTAATGGGAGAATCATGGACTGTGCTTTTACTGTGGCTTTTAATCCTCAATTTGAACAACTACTTTTAGCTAGCAAAGAAGCTACTTACGCTGGAGTTAAAGCTTTAGGTGTTGATGTAAGATTATGTGAAATAGGAAGAGACATTCATGAAGTGATGAAGAGCTTTGAAGTTCAGATTGATGGAGTTACTTACCCTATAAAACCTATTTATGATTTACATGGTCATAGCATTTCTCAGTATACAATACACGCCGGGCAGTCTATACCTTGTTATGATAACGGGGATACTACAAGAATTAAGGAAAATACTTTCTATGCTGTAGAAACTTTTGCTTCTACAGGTAAAGGTCGAATTTCTGATAAATCGCCTTGTACTCATTACATTTTGAACAAAAACAAACAGAGAAAATTATTTGACAAAAACTGTATTGCTGTCTATAATTTTATAAAAGATAATTTAGGAACTCTACCCTTTAGTCCTAAACATATTGATCATTATGGAATTATCAAACTTCCCTCTTACACATACATTAAGATGCTTACTATGATGGGTCTTATTACCCCTTATCCTCCCCTTAATGATATTAAAGGATCGTATGTTGCACAATTTGAACATACAATTTATGTTACAGAAAACGGTAAAGAAATTCTTACAAAAGGAGATGATTTTTAATAAAATTTTAACAAAAGAGGAGTTTGTTGTATTTTTTATAATTTAATGAAAAGTTTATGTGGTTATCTACCGTCGAAATAGATCGATTTATTTTTTAAATTTAAAGTATCCGTGTTCAATAATATGAAGGTTTTTTTTTTTTTTTTTTTTTT
