# Supplementary material for: Establishment and application of a loop-mediated isothermal amplification method based on MetAP2 gene for the detection of Nosema bombycis in silkworms (Bombyx mori)
Source: Front Vet Sci. 2025 Mar 10;12:1549224. doi: 10.3389/fvets.2025.1549224 (PMC11931649; doi:10.3389/fvets.2025.1549224)
Supplement: Supplementary file 2 [file Table_2.docx]

Supplementary Table 1 LAMP primer sets based on *MetAP2* gene of *Nosema bombycis*

| **Primer set name** | **Position** | **Primer sequence** | **Product length (bp)** | **GC rate** | **Tm (C) predicted** |
| --- | --- | --- | --- | --- | --- |
| LM 1 | F3: 5'pos: 458; 3'pos: 476  B3: 5'pos: 630; 3'pos: 651  F2: 5'pos: 477; 3'pos: 499  F1c:5'pos: 518; 3'pos: 542  B2: 5'pos: 604; 3'pos: 625  B1c:5'pos: 543; 3'pos: 567 | F3:5’ GGGAGAATCATGGACTGTG 3’  B3:5’ ATAGGGTAAGTAACTCCATCAA 3’  FIP: 5’ CGTAAGTAGCTTCTTTGCTAGCTAA-CTTTTACTGTGGCTTTTAATCCT 3’  BIP: 5’ CTGGAGTTAAAGCTTTAGGTGTTGA-AACTTCAAAGCTCTTCATCACT 3’ | 194 | F3: 0.53  B3: 0.36 | F: 56.56  B3: 55.57 |
| LM 2 | F3: 5'pos: 430; 3'pos: 452  B3: 5'pos: 618; 3'pos: 638  F2: 5'pos: 453; 3'pos: 473  F1c:5'pos: 511; 3'pos: 535  B2: 5'pos: 595; 3'pos: 616  B1c:5'pos: 537; 3'pos: 561 | F3:5’AAAAATAGATTTTGGGACACATG 3’  B3:5’ CTCCATCAATCTGAACTTCAA 3’  FIP: 5’ AGCTTCTTTGCTAGCTAAAAGTAGT-TTAATGGGAGAATCATGGACT3’  BIP: 5’CTTACGCTGGAGTTAAAGCTTTAGG-GCTCTTCATCACTTCATGAATG 3’ | 209 | F3: 0.30  B3: 0.38 | F: 55.43  B3: 55.13 |
| LM 3 | F3: 5'pos: 520; 3'pos: 538  B3: 5'pos: 705; 3'pos: 722  F2: 5'pos: 542; 3'pos: 562  F1c:5'pos: 587; 3'pos: 611  B2: 5'pos: 670; 3'pos: 690  B1c:5'pos: 612; 3'pos: 636 | F3:5’AGCTAGCAAAGAAGCTACT3’  B3:5’AAGGTATAGACTGCCCGG3’  FIP: 5’ TCATCACTTCATGAATGTCTCTTCC-GCTGGAGTTAAAGCTTTAGGT3’  BIP: 5’AGAGCTTTGAAGTTCAGATTGATGG-TGAGAAATGCTATGACCATGT3’ | 203 | F3: 0.42  B3: 0.56 | F: 55.13  B3: 57.06 |
| LM 4 | F3: 5'pos: 587; 3'pos: 607  B3: 5'pos: 776; 3'pos: 796  F2: 5'pos: 608; 3'pos: 628  F1c:5'pos: 668; 3'pos: 692  B2: 5'pos: 752; 3'pos: 774  B1c:5'pos: 698; 3'pos: 717 | F3:5’GGAAGAGACATTCATGAAGTG3’  B3:5’ ACCTGTAGAAGCAAAAGTTTC3’  FIP: 5’ ACTGAGAAATGCTATGACCATGTAA-ATGAAGAGCTTTGAAGTTCAG3’  BIP: 5’ATACACGCCGGGCAGTCTAT-ACAGCATAGAAAGTATTTTCCTT3’ | 210 | F3: 0.43  B3: 0.38 | F: 55.78  B3: 55.88 |
| LM 5 | F3: 5'pos: 369; 3'pos: 386  B3: 5'pos: 557; 3'pos: 581  F2: 5'pos: 387; 3'pos: 406  F1c:5'pos: 437; 3'pos: 461  B2: 5'pos: 526; 3'pos: 545  B1c:5'pos: 462; 3'pos: 486 | F3:5’CTGCTCATTTTACCGTGC3’  B3:5’CACATAATCTTACATCAACACCTAA3’  FIP: 5’ TCCCATTAACATGTGTCCCAAAATC-TTCCAGACGATAACACTACG3’  BIP: 5’GAATCATGGACTGTGCTTTTACTGT-CAGCGTAAGTAGCTTCTTTG3’ | 213 | F3: 0.50  B3: 0.32 | F: 55.46  B3: 56.79 |

Supplementary Table 2 Sequences of Universal and specific LAMP primers used for comparison

| **Primer (gene) name** | **Primer sequence** | **Remarks** |
| --- | --- | --- |
| Universal primer  (16s rDNA/*ssu rRNA*) | F3: GGGGATAGTATGATCGCAAG  B3: CCTCTCCTTCATATGTATCACTAC  FIP: ACCCCGGGTTGAGTCAAATTAA-AAAGTGACGGAAGAATACCAC  BIP: GTGCATGGCCGTTTCCAATG-AGGGTCTCACATCTTGTTG | This LAMP primer was based on gene sequences of a number of microsporidian species |
| Specific primer (*Septin3*) | F3: ACACATTTAGGAAAAGTGCTTTA  B3: AATTTGTTACAGAAGGACTCC  FIP: CTCTCTTATCACGTTTTCCATTTCA-AAGGGAAAAGAGCTCGGA  BIP: TGAGGATTTGAAACGGGAAGAACAT-AATACTACCACCCACCGA | This LAMP primer was specifically based on sequence of *septin3 gene of Nosema bombycis* |

Note: These LAMP primers are part of our unpublished results.
