# Supplementary material for: Establishment and application of a loop-mediated isothermal amplification method based on MetAP2 gene for the detection of Nosema bombycis in silkworms (Bombyx mori)
Source: Front Vet Sci. 2025 Mar 10;12:1549224. doi: 10.3389/fvets.2025.1549224 (PMC11931649; doi:10.3389/fvets.2025.1549224)
Supplement: Supplementary file 3 [file Table_3.docx]

**Establishment and Application of a Loop-Mediated Isothermal Amplification Method Based on *MetAP2* Gene for the Detection of *Nosema bombycis* in Silkworms (*Bombyx mori*)**

Izhar Hyder Qazi^1*^, Ting Yuan^1^, Sijia Yang^1^, Christiana Angel^2, 3^, Jiping Liu^1^*

^1^Guangdong Provincial Key Lab of Agro-Animal Genomics and Molecular Breeding, College of Animal Science, South China Agricultural University, Guangzhou 510642, Guangdong, China

^2^Key Laboratory for Agro-Ecological Processes in Subtropical Region, Institute of Subtropical Agriculture, The Chinese Academy of Sciences, Changsha, China; the University of Chinese Academy of Sciences, Beijing, China

^3^Shaheed Benazir Bhutto University of Veterinary and Animal Sciences, Sakrand, Pakistan

*Corresponding authors: IHQ (vetdr_izhar@yahoo.com); JL (liujiping@scau.edu.cn)

### Construction of MetAP2-pMD19T plasmid standard

The PCR amplification conditions were: 94°C for 5 min; 32 cycles of 94°C for 30 s, 53°C for 45 s, 72°C for 45 s; and 72°C for 10 min. Following detection of the PCR amplification product by agarose gel electrophoresis, 40 μL of the PCR product containing the target fragment was selected. 1.2% agarose gel (EB staining) electrophoresis was used to detect and recover the specific target fragment under UV light. The recovery of the target DNA fragment from the agarose gel was carried out using TaKaRa MiniBEST DNA Gel Recovery Kit as per the manufacturer’s instructions. The recovered target fragment was ligated to the pMD™19-T vector (ligation system: 1 μL of PCR recovery product, 1 μL of pMD-19T vector, 3 μL of dH2O, 5 μL of Solution I), mixed thoroughly and ligated at 16°C for 1 h. The ligation product was transferred into competent cells as follows: The competent cells were taken out of the -70°C refrigerator and placed on ice for thawing. Then, the ligation product was added, incubated on ice for 30 min, heat shocked at 42°C for 90 sec, and then quickly placed on ice and incubated for 2 min. Then sterile LB medium (without ampicillin) was added to each tube in the clean workbench to make the final volume of 1 mL. The mixture was incubated for 1 h in a shaker (150 r/min) at 37°C. Then, 100 μL of liquid was taken and spread evenly on the LB plate containing ampicillin. The plates were placed upside down in a 37°C constant-temperature incubator and incubated for 12 to 16 h. White colonies of appropriate size were picked up by using a sterile pipette tips and put into a test tube containing 2 mL of ampicillin-resistant LB liquid culture medium, and incubated at 37°C with shaking at 200 r/min for 12 h. When the bacterial cells become turbid, the bacterial solution was transferred to a 1.5 mL centrifuge tube. The plasmid extraction was performed according instructions of the TaKaRa MiniBEST plasmid purification kit manual. The PCR verification was done to confirm whether the size of the inserted fragment in the vector was consistent with the target fragment. The amplified fragments were selected and sent to Sangon Biotechnology Co., Ltd., Shanghai, China for sequencing and identification.

**Fumagillin Treatment**

Group 4A was continuously fed normal mulberry leaves from the 4th instar to mature stages. Group 4B was fed normal mulberry leaves treated only with fumagillin from the 4th instar to mature stages. Group 4C was initially fed mulberry leaves artificially contaminated with *Nosema bombycis* spores (10^5^ spores/mL) for 24 h. These silkworms were then fed mulberry leaves treated with fumagillin solution continuously from the 4th instar to mature stages. Group 4D was fed only mulberry leaves artificially contaminated with *Nosema bombycis* spores (10^5^ spores/mL) from the 4th instar to mature stages (Silkworms were fed mulberry leaves artificially contaminated with *Nosema bombycis* spores once a day and normal mulberry leaves twice a day).

Silkworms in group 5A were continuously fed normal mulberry leaves from the 5th instar to mature stages. Group 5B was fed normal mulberry leaves treated only with fumagillin from the 5th instar to mature stages. Group 5C was initially fed mulberry leaves artificially contaminated with *Nosema bombycis* spores (10^5^ spores/mL) for 24 h. These silkworms were then fed mulberry leaves treated with fumagillin solution continuously from the 5th instar to mature stages. Group 5D was fed only mulberry leaves artificially contaminated with *Nosema bombycis* spores (10^5^ spores/mL) from the 5th instar to mature stages (Silkworms were fed mulberry leaves artificially contaminated with *Nosema bombycis* spores once a day and normal mulberry leaves twice a day).
