# Supplementary material for: Establishment and application of a loop-mediated isothermal amplification method based on MetAP2 gene for the detection of Nosema bombycis in silkworms (Bombyx mori)
Source: Front Vet Sci. 2025 Mar 10;12:1549224. doi: 10.3389/fvets.2025.1549224 (PMC11931649; doi:10.3389/fvets.2025.1549224)
Supplement: Supplementary file 4 [file Data_Sheet_1.docx]

**Establishment and Application of a Loop-Mediated Isothermal Amplification Method Based on *MetAP2* Gene for the Detection of *Nosema bombycis* in Silkworms (*Bombyx mori*)**

Izhar Hyder Qazi^1*^, Ting Yuan^1^, Sijia Yang^1^, Christiana Angel^2, 3^, Jiping Liu^1^*

^1^Guangdong Provincial Key Lab of Agro-Animal Genomics and Molecular Breeding, College of Animal Science, South China Agricultural University, Guangzhou 510642, Guangdong, China

^2^Key Laboratory for Agro-Ecological Processes in Subtropical Region, Institute of Subtropical Agriculture, The Chinese Academy of Sciences, Changsha, China; the University of Chinese Academy of Sciences, Beijing, China

^3^Shaheed Benazir Bhutto University of Veterinary and Animal Sciences, Sakrand, Pakistan

*Corresponding authors: IHQ (vetdr_izhar@yahoo.com); JL (liujiping@scau.edu.cn)

**Supplementary Figures**


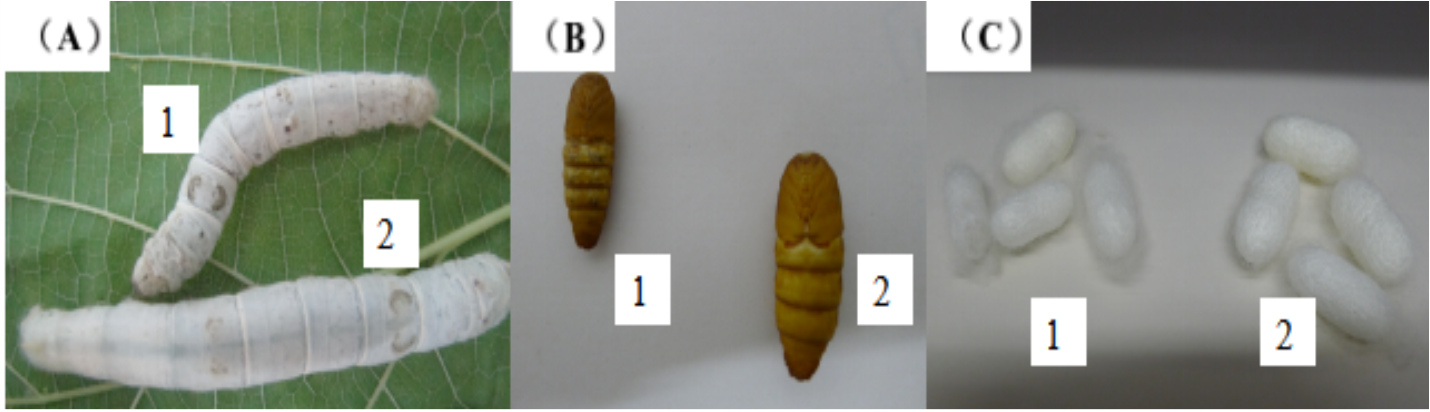


**Figure S1 Representative images showing gross morphological comparison of silkworms infected with *Nosema bombycis* before and after treatment with fumagillin.**

Note: In panel A, 1 is a silkworm infected with *Nosema bombycis*, and 2 is a silkworm infected with *Nosema bombycis* and then treated with fumagillin.

In panel B, 1 is a silkworm pupa infected with *Nosema bombycis*, and 2 is a silkworm pupa infected with *Nosema bombycis* and then treated with fumagillin.

In panel C, 1 is a cocoon infected with *Nosema bombycis*, and 2 is a cocoon infected with *Nosema bombycis* and then treated with fumagillin.


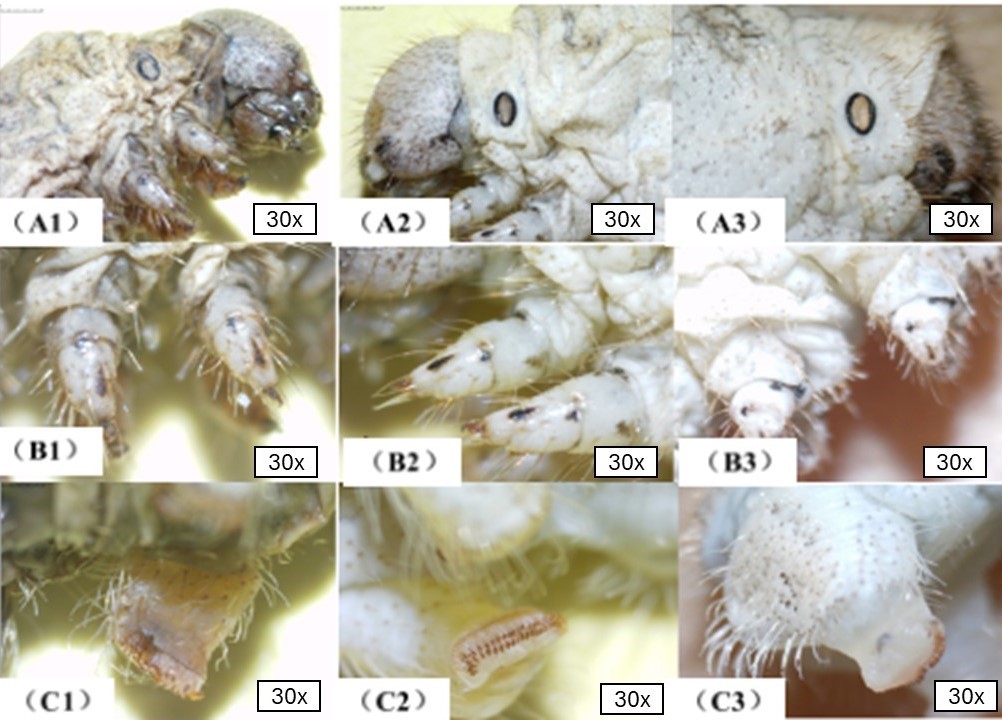


**Figure S2** Representative stereomicroscopic images showing morphological features of body parts of silkworms in different groups (30×)

Note: A1. Head of infected silkworm. A2. Head of a healthy silkworm. A3. Head of silkworm treated with fumagillin.

B1. Abdominal legs of infected silkworm. B2. The abdominal legs of healthy silkworm. B3. The abdominal legs of silkworm treated with fumagillin. C1. Uropod of infected silkworm. C2. The uropod of healthy silkworm. C3.The uropod of silkworm treated with. For detailed description, please see the main text file.


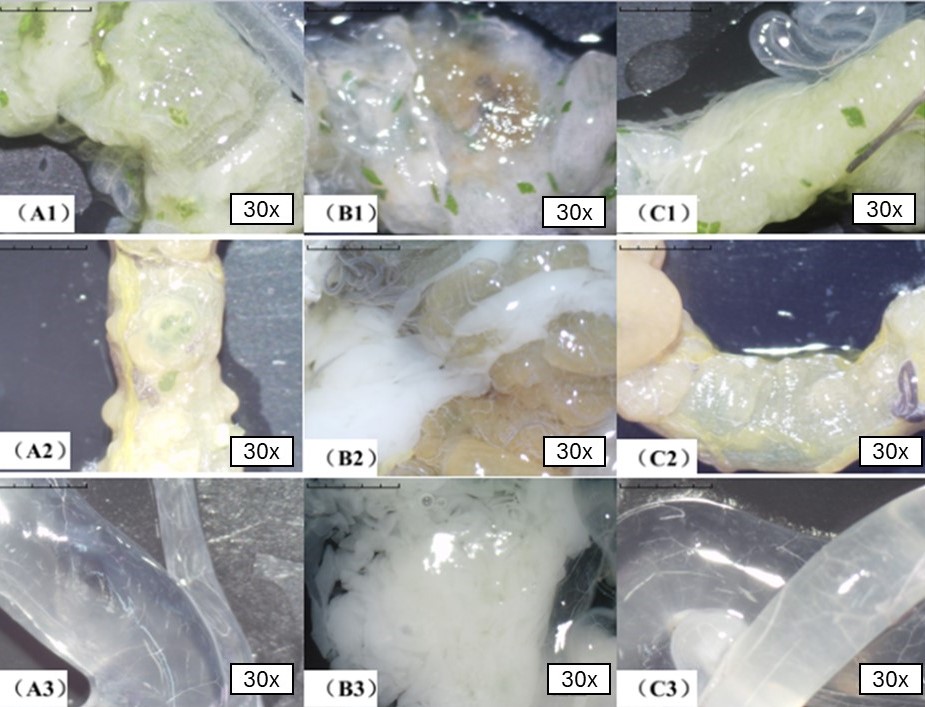


**Figure S3** Representative stereomicroscopic images showing midguts and silk glands of healthy, infected, and infected + fumagillin-treated silkworms * (30×)

Note: * addition of fumagillin six h after 24 h of exposure to spores).

Figure A1 shows the normal midgut and silk glands of silkworms at 30 h. Figure B1 shows the midgut and silk glands of silkworms infected with *Nosema bombycis* spores at 30 h. Figure C1 shows the midgut and silk glands of silkworms infected *Nosema bombycis* spores and then treated with fumagillin at 30 h.

Figure A2 shows the midgut of a normal silkworm at 30 h, Figure B2 shows the midgut of a silkworm infected with *Nosema bombycis* at 30 h.

Figure A3 shows the normal silk gland of silkworm at 30 h, Figure B3 shows the silk gland of silkworm infected with *Nosema bombycis* spores at 30 h, Figure C3 shows the silk gland of silkworm infected with *Nosema bombycis* spores and then treated with fumagillin at 30 h.

For detailed description, please see the main text file.
